# Complementing conventional infectious disease surveillance with national health insurance claims data in the Republic of Korea

**DOI:** 10.1038/s41598-019-45409-3

**Published:** 2019-06-19

**Authors:** Jaehun Jung, Jae Hyoung Im, Young-Jin Ko, Kyungmin Huh, Chang-gyo Yoon, Chulwoo Rhee, Young-Eun Kim, Dun-Sol Go, Arim Kim, Yunsun Jung, Munkhzul Radnaabaatar, Seok-Jun Yoon

**Affiliations:** 1 Department of Preventive Medicine, Gachon University College of Medicine, Incheon, Korea; 2 Department of Infectious Diseases, Inha University School of Medicine, Incheon, Republic of Korea; 3 Department of Preventive Medicine, Seoul National University College of Medicine, Seoul, Republic of Korea; 4 Department of Infectious Diseases, Samsung Medical Center, Sungkyunkwan University School of Medicine, Seoul, Republic of Korea; 5 Preventive Medicine Program, Graduate School of Public Health, Seoul National University, Seoul, Korea; 6 Department of Preventive Medicine, Korea University College of Medicine, Seoul, Republic of Korea

**Keywords:** Epidemiology, Epidemiology

## Abstract

Surveillance remains an important tool for timely outbreak detection and response. Many countries, including Korea, have established national infectious disease surveillance systems with clinical notification. We aimed to evaluate the National Health Insurance Claims-based Surveillance (NHICS) compared to conventional passive report-based National Infectious Diseases Surveillance (NIDS). Reported to claimed cases ratios (R/C ratio) were evaluated from monthly notifiable disease cases captured by NIDS and NHICS. The relationships between 26 infectious diseases and each surveillance system were analysed using Pearson’s correlation analysis and linear regression. There was an overall increase in R/C ratio from 2010–2017 (0.37 to 0.78). In 22 infectious diseases, there was a correlation between NIDS and NHICS. Moreover, claim-based surveillance showed less fluctuating disease incidence rates than report-based surveillance for specific infectious diseases, such as varicella, mumps, and scarlet fever. However, for infectious diseases with episodic outbreaks or low incidence, it was difficult to assess NHICS usefulness. Claim-based surveillance is less affected by limitations of conventional report-based surveillance systems, such as reporting rate. Given delays in claim systems, a claim-based surveillance is expected to be complementary to conventional systems for the detection of various infectious diseases with the advancement of bio-information technology.

## Introduction

There has been an increase in international public health emergencies by newly recognised or re-emerging infectious diseases due to mobile populations, climate, and socio-environmental interactions^1^. Given that monitoring infectious diseases is critical to the control and prevention of epidemics, the establishment and implementation of robust and sensitive disease surveillance systems is needed to ensure early detection of epidemics.

Most countries operate disease surveillance systems based on active or passive reporting according to priorities^2^. Active surveillance systems can provide relatively accurate and prompt information on infectious diseases of concern, but usually cover a limited range of diseases due to cost and time considerations^2^. Thus, many countries rely on passive surveillance systems with clinical notifications for monitoring the majority of infectious diseases. Compared with active systems, passive systems enable continuous monitoring over large geographical areas at relatively lower cost. However, incomplete data may be collected in a passive surveillance system, with low reporting rates requiring additional investigations or data collection processes^3^.

In the Republic of Korea (ROK), the 2015 Middle East respiratory syndrome coronavirus (MERS-CoV) infection outbreak demonstrated the failure of national surveillance systems in detecting the outbreak in early phases to warn the public^4,5^. The government enhanced human resources and financial support for strengthened surveillance, with recognition of the importance of vigilant disease surveillance systems^6^.

Many countries have developed and operated complementary surveillance systems to improve passive surveillance systems^7–9^. Recently, surveillance systems using medical records, such as health insurance claim data^10,11^ and drug utilisation review^12,13^ have been introduced, because many countries with health insurance systems have established health information databases in operating insurance schemes. The National Health Insurance Service of the ROK has been collaborating with the Health Insurance Review and Assessment Service (HIRA), that have managed electronic health insurance claims data reported by all medical service providers since 2001^14^. A number of studies have tested the benefit of claim-based surveillance systems after the National Health Insurance Service merged into a single payer scheme in 2001^15,16^.

In this study, we sought to compare the performance of the conventional report-based National Infectious Diseases Surveillance (NIDS) and National Health Insurance Claims-based Surveillance (NHICS) to assess its usefulness and applicability. We conclude with how health insurance claim data can be best used to monitor disease activities.

## Methods

### Study period and target diseases

During January 2010 to June 2017, we analysed NIDS and NHICS data to compare the number of national notifiable infectious disease cases of 80 infectious diseases^17^. Of these, 46 are been monitored for the possibility of imported or re-emerging cases; the diseases had not been steadily reported in the ROK. Therefore, these 46 diseases were excluded from this study. Among the remaining infectious diseases, those with less than ten patients per year in the NIDS and NHICS were excluded. Additionally, diseases that were included in the NIDS since 2013 (such as MERS-CoV, Zika virus, and hepatitis C virus) were excluded for comparability. Chronic infectious diseases, such as hepatitis B virus, syphilis, leprosy, and tuberculosis were also excluded due to possibility of duplicated case numbers and complex diagnostic codes. Human immunodeficiency virus/acquired immunodeficiency syndrome (HIV/AIDS) infection is not included in NIDS due to the special protection act for HIV/AIDS patients^18^, and was excluded. Finally, data of 26 infectious diseases were analysed and the International Statistical Classification of Disease and Related Health Problems, 10th Revision (ICD-10) codes of target infectious diseases are shown in Supplementary Table 1.

### Data sources

We assessed the number of patients imported from the NIDS web reporting system (http://is.cdc.go.kr) in order to compare that with the surveillance performance of the NHICS. The standards for NIDS include confirmed and suspected cases; however, viral hepatitis A (HAV) and malaria are reported to the NIDS after confirmation. It is a legal duty for physicians to report infectious diseases using the online reporting system when diagnosis is made.

**Figure 1:**
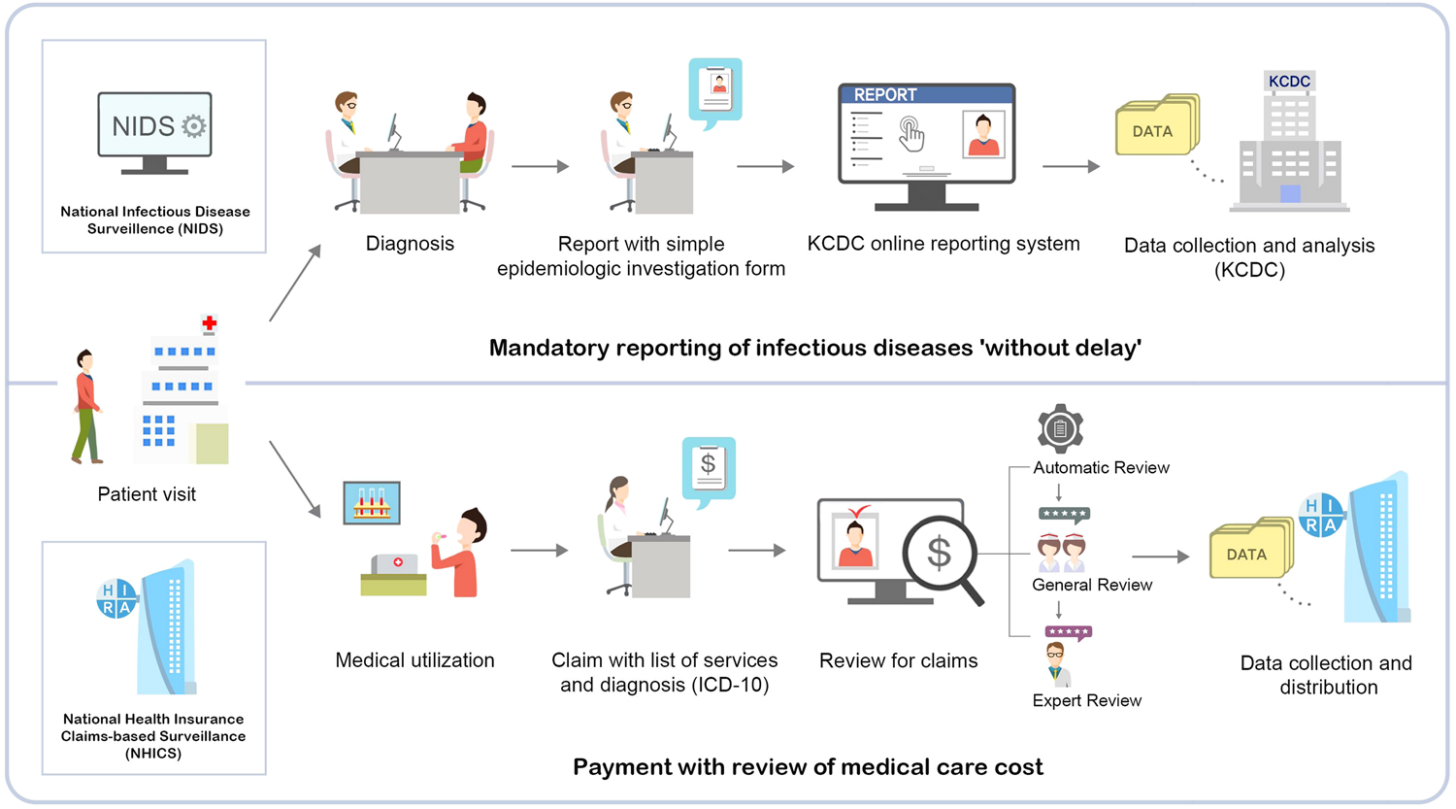
A Framework of National Infectious Diseases Surveillance (NIDS) and National Health Insurance Claims-based Surveillance (NHICS) in Republic of Korea.

To analyse the claim-based surveillance data, we retrieved health insurance data from the HIRA, which provides de-identified data after the review of claims. Since all medical service providers must contract with the National Health Insurance Service as a social health insurance scheme, it reimburses all medical costs according to diagnostic codes, based on ICD-10^19^. When a medical provider claims for reimbursement, HIRA takes 3 steps in reviewing the claim: automatic, general, and expert. Figure 1 shows the reporting system and data processing steps of the surveillance systems.

### R/C ratio

In report-based surveillance, reported cases (R) can be generated using the following formula:

$${Re}ported\,Cases\,(R)=incident\,cases\,(I)\times possibility\,of\,visiting\,physician\,(P)\times reporting\,rate\,(RR)$$

In claim-based surveillance, claimed cases (C) can be reported using the following formula:

$$Claimed\,cases\,(C)=incident\,cases\,(I)\times possibility\,of\,medical\,utilisation\,(P)\times claim\,rate\,(CR).$$

CR is nearly 100% in the ROK; the ratio of reported/claimed cases (R/C ratio) can be interpreted as an indicator for the reporting rate (RR) of NIDS, with the assumption that the diagnostic accuracies are the same.

### Interrupted time series analysis of overall R/C ratio

We conducted an interrupted time series (ITS)^20^ to evaluate the changes in the R/C ratio over time and analysed the effect of enhanced report-based surveillance after the 2015 MERS-CoV infection outbreak. The ITS provides a tracking function on the time trend of R/C ratio, and the change in trend and level change after June 2015.

### Correlation between surveillance systems

Pearson’s correlation analysis was employed to demonstrate the correlation between reported and claimed cases in both systems, and linear regression analysis was conducted to identify changes in the reporting rate over time and the fitness of the linear model. Two-tailed *P*-values of <0.05 were considered statistically significant. All statistical analyses were performed using SAS 9.4 (SAS Institute Inc., Cary, NC, USA).

### Ethical consideration

The study protocol was approved by the institutional review board of Korea University (approval number: KU-IRB-18-EX-51-A-1) and was performed in accordance with the relevant guidelines and regulations. As this study used secondary data obtained from the NHICS and NIDS and contained no personal information, the need for informed consent was waived.

## Results

We analysed 26 infectious diseases using the NIDS and NHICS. We analysed 22 diseases of interest for 90 months of the surveillance period, and the other 4 infectious diseases presented different time frames due to a change in the Infectious Disease Control and Prevention Act since January 2010: Creutzfeldt-Jakob disease (CJD), HAV, Lyme disease, and severe fever with thrombocytopenia syndrome (SFTS). The list of the national notifiable diseases investigated, number of reported and claimed cases, and observation period are presented in Table 1. The number of claimed cases was more than that reported for the 24 infectious diseases, except enterohemorrhagic *Escherichia coli* (EHEC) infection and legionellosis. The total R/C ratio of the 26 infectious diseases was 0.43. Given that the ideal R/C ratio is 1.0, the average ratio for cholera was 0.02 and 0.03 for rubella. On the other hand, the R/C ratio of legionellosis was 2.11, and that of EHEC infection was 1.56. The overall annual R/C ratio increased sharply from 0.37 in 2010 to 0.78 in 2017 (Table 2).

**Table 1:** Reported cases and claimed cases of 26 infectious disease in Republic of Korea, Jan 2010–June 2017.

Diseases	Surveillance Period (Months)	Reported Cases (Monthly Average)	Claimed Cases (Monthly average)	Monthly Average R/C Ratio^a^
Brucellosis	Jan 2010–Jun 2017 (90)	102 (1.1)	342 (3.8)	0.319
Cholera	Jan 2010–Jun 2017 (90)	21 (0.2)	2,925 (32.5)	0.016
Creutzfeldt-Jakob disease (CJD)	Jan 2011–Jun 2017 (78)	266 (3.4)	2,311 (29.6)	0.117
Dengue fever	Jan 2010–Jun 2017 (90)	1,388 (15.4)	1,706 (19.0)	0.757
Enterohaemorrhagic Escherichia coli (EHEC)	Jan 2010–Jun 2017 (90)	581 (6.5)	405 (4.5)	1.563
Viral hepatitis A	Jan 2011–Jun 2017 (78)	18,100 (232.1)	81,844 (1,049.3)	0.221
Haemorrhagic fever with renal syndrome (HFRS)	Jan 2010–Jun 2017 (90)	3,209 (35.7)	4,999 (55.5)	0.595
Japanese encephalitis	Jan 2010–Jun 2017 (90)	158 (1.8)	612 (6.8)	0.174
Legionellosis	Jan 2010–Jun 2017 (90)	377 (4.2)	249 (2.8)	2.109
Leptospirosis	Jan 2010–Jun 2017 (90)	492 (5.5)	1,040 (11.6)	0.537
Lyme disease	Jan 2011–Jun 2017 (78)	77 (1.0)	304 (3.9)	0.344
Malaria	Jan 2010–Jun 2017 (90)	5,743 (63.8)	18,624 (206.9)	0.239
Measles	Jan 2010–Jun 2017 (90)	737 (8.2)	4,166 (46.3)	0.089
Meningococcal meningitis	Jan 2010–Jun 2017 (90)	59 (0.7)	118 (1.3)	0.477
Mumps	Jan 2010–Jun 2017 (90)	111,377 (1,237.5)	224,556 (2,495.1)	0.494
Murine typhus	Jan 2010–Jun 2017 (90)	186 (2.1)	326 (3.6)	0.727
Paratyphoid fever	Jan 2010–Jun 2017 (90)	392 (4.4)	1,141 (12.7)	0.546
Pertussis	Jan 2010–Jun 2017 (90)	901 (10.0)	4,902 (54.5)	0.375
Q fever	Jan 2010–Jun 2017 (90)	201 (2.2)	466 (5.2)	0.384
Rubella	Jan 2010–Jun 2017 (90)	178 (2.0)	5,337 (59.3)	0.031
Scarlet fever	Jan 2010–Jun 2017 (90)	43,043 (478.3)	97,096 (1,078.8)	0.396
Severe fever with thrombocytopenia (SFTS)	Jul 2013–Jun 2017 (48)	343 (7.1)	1,381 (28.8)	0.215
Shigellosis	Jan 2010–Jun 2017 (90)	1,147 (12.7)	6,537 (72.6)	0.201
Scrub typhus	Jan 2010–Jun 2017 (90)	59,087 (656.5)	99,577 (1,106.4)	0.408
Typhoid fever	Jan 2010–Jun 2017 (90)	1,121 (12.5)	2,704 (30.0)	0.441
Varicella	Jan 2010–Jun 2017 (90)	307,886 (3,421.0)	957,569 (10,639.7)	0.364

^a^In cases where it was impossible to calculate the monthly R/C ratio due to the absence of claimed case, it was assumed that 0.9 cases were claimed.

**Table 2:** Annual reported cases per claimed cases ratio (R/C ratio) of 26 infectious diseases, 2010–2017.

	2010^a^	2011^b^	2012^b^	2013	2014	2015	2016	2017^c^
Annual Average R/C ratio (Standard Deviation)	0.37 (0.30)	0.26 (0.24)	0.35 (0.30)	0.40 (0.34)	0.44 (0.38)	0.54 (0.45)	0.67 (0.63)	0.78 (0.91)

^a^Creutzfeldt-Jakob disease, Lyme disease, Severe fever with thrombocytopenia syndrome (SFTS), Viral hepatitis A were not included.

^b^SFTS was not included.

^c^Jan 2017–Jun 2017.

### Monthly reported/claimed cases and R/C ratio

**Figure 2:**
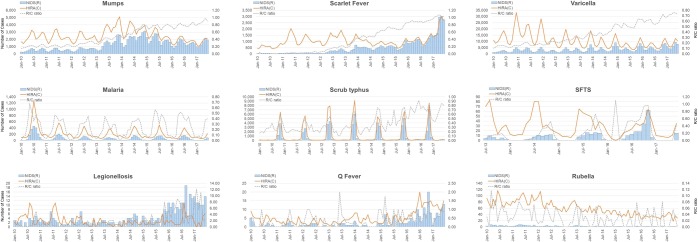
Trend of 9 infectious diseases’ reported, claimed cases, and reported cases per claimed cases ratio (R/C ratio) in Republic of Korea, Jan 2010–Jun 2017.

We compared the monthly reported/claimed cases and monthly R/C ratios of the 2 surveillance systems based on the 26 infectious diseases. Figure 2 demonstrates 9 infectious diseases; the remaining 17 diseases are shown in Supplementary Fig. 1. Scarlet fever, mumps, and varicella presented a sharp rise in R/C ratio, with the graphs of the monthly cases of the two systems almost identical over time. Particularly, scarlet fever showed a dramatic rise in R/C ratio and varicella presented a decreasing trend of cases in the claim-based surveillance, although an increase was observed in the report-based surveillance, with relatively fast rising R/C ratio. Dengue fever and murine typhus showed a good concordance (R/C ratio between 0.7–1.0) of the 2 surveillance systems, although baseline noises were observed in the claim data. Diseases such as rubella, Q fever, and legionella showed a large R/C ratio variation over time.

### ITS analysis of R/C ratio

**Figure 3:**
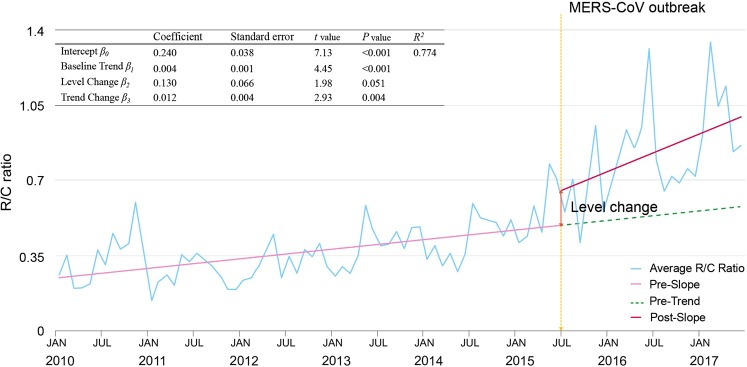
Interrupted time series analysis of reported cases per claimed cases ratio (R/C ratio) after Middle East respiratory syndrome coronavirus outbreak in Republic of Korea, Jan 2010–Jun 2017.

The R/C ratios of all infectious diseases were an upward trend (Fig. 2). Therefore, we performed ITS analysis to evaluate the effect of MERS-CoV outbreak on the increased R/C ratio. In the ITS analysis, although the overall R/C ratio of infectious diseases throughout the study period was relatively steadily increasing, there was an abrupt trend and level change in the R/C ratio during June 2015 (Fig. 3).

### Correlation between report-based surveillance and claim-based surveillance

In the overall Pearson’s R values, 22 diseases had a statistically significant correlation between the systems, and after the MERS-CoV outbreak 19 diseases showed a correlation between claimed and reported cases, despite the relatively short observation period. Particularly, high overall Pearson R values were recorded for scrub typhus (0.98), malaria (0.95), measles (0.92), HFRS (0.91), and dengue fever (0.87). However, in rare infectious diseases, such as brucellosis, CJD, meningococcaemia, and rubella, or sporadic outbreaks e.g., legionella and cholera, a statistical relationship between the surveillance systems was not observed. We performed a linear regression analysis to observe changes over time and fitness of the linear model. The majority of diseases showed statistically significant linearity, except for Japanese encephalitis and murine typhus. Varicella, scarlet fever, and mumps demonstrated high R^2^ values (0.93, 0.93, and 0.92, respectively); HAV presented a considerably high R^2^ value (0.61). In contrast, HFRS (0.31), leptospirosis (0.23), SFTS (0.10), and malaria (0.001) recorded low R^2^ values. Legionellosis, scarlet fever, EHEC, leptospirosis, and Lyme disease had beta coefficients (slopes of linear regression model) greater than 0.01, indicating a rapid increase in R/C ratio over time (Table 3).

**Table 3:** Pearson correlation between report-based surveillance and claim-based surveillance and linear regression analysis of reported cases per claimed cases ratio (R/C ratio) by months.

Diseases	Pearson correlation between report- based and claim-based surveillance system				Linear regression of monthly R/C ratio	
	Overall period		Post-2015 MERS-CoV outbreak		*β*	*R* ^2^
	*R*	*P* value	*R*	*P* value		
Brucellosis	0.59	<0.01	−0.08	0.71	0.0000	0.000
Cholera	0.02	0.84	0.08	0.71	0.0002	0.011
Creutzfeldt-Jakob disease (CJD)	0.26	0.02	0.15	0.47	−0.0007	0.034
Dengue fever	0.87	<0.01	0.76	<0.01	0.0056	0.196
Enterohaemorrhagic Escherichia coli (EHEC)	0.73	<0.01	0.67	<0.01	0.0186	0.110
Viral hepatitis A	0.76	<0.01	0.93	<0.01	0.0044	0.614
Haemorrhagic fever with renal syndrome (HFRS)	0.91	<0.01	0.95	<0.01	0.0056	0.313
Japanese encephalitis	0.66	<0.01	0.74	<0.01	−0.0006	0.002
Legionellosis	0.20	0.06	0.21	0.33	0.0520	0.315
Leptospirosis	0.78	0.00	0.94	<0.01	0.0119	0.229
Lyme disease	0.23	0.04	0.52	0.01	0.0127	0.282
Malaria	0.95	<0.01	0.94	<0.01	0.0002	0.001
Measles	0.92	<0.01	0.38	0.07	−0.0006	0.008
Meningococcal meningitis	0.19	0.08	0.16	0.46	0.0062	0.040
Mumps	0.63	<0.01	0.94	<0.01	0.0094	0.916
Murine typhus	0.68	<0.01	0.59	<0.01	−0.0021	0.005
Paratyphoid fever	0.28	0.01	0.63	<0.01	0.0123	0.416
Pertussis	0.12	0.26	0.89	<0.01	0.0089	0.437
Q fever	0.73	<0.01	0.58	<0.01	0.0051	0.099
Rubella	0.59	<0.01	0.05	0.83	−0.0004	0.125
Scarlet fever	0.66	<0.01	1.00	<0.01	0.0135	0.929
Severe fever with thrombocytopenia (SFTS)	0.54	<0.01	0.80	<0.01	0.0064	0.101
Shigellosis	0.43	<0.01	0.59	<0.01	0.0027	0.216
Scrub typhus	0.98	<0.01	0.99	<0.01	0.0054	0.453
Typhoid fever	0.67	<0.01	0.68	<0.01	0.0035	0.227
Varicella	0.36	<0.01	0.98	<0.01	0.0073	0.931

## Discussion

Accuracy and responsiveness are key attributes of robust report-based surveillance systems. While evaluating reporting rates requires effort and time, the ratio of reporting cases to claim cases can be considered as a convenient indicator of the reporting rate^21^. We used R/C ratio as an indicator of reporting rate. Additionally, we used the R/C ratio to compare the performance of two surveillance systems, demonstrating that various factors may affect performance. We found that the overall ratio increased in most infectious diseases after the MERS-CoV outbreak, which may have promoted disease reporting from health service providers and the general awareness of public on notifiable diseases^22^. This may have strengthened the performance of the national disease surveillance system. However, an increase in the R/C ratio may be affected by unexpected factors. For example, patients suspected of having scarlet fever were included in the reporting standard since September 2012. Moreover, private complementary health insurance schemes have become very popular in the ROK; patients have asked doctors to issue certificates that clinicians have reported to NIDS as a requirement for reimbursement from private insurance^23^. This might have led to an increase in the reporting rate and R/C ratio. Such phenomenon may cause the misinterpretation of other infectious diseases (e.g., varicella) if only report-based surveillance is used^24,25^. Furthermore, the R/C ratio for legionellosis and EHEC far exceeded 1. This means that some patients were notified without proper diagnosis codes. These diseases were often confirmed after the patient was discharged in the past; however, after MERS, the reporting system reform led to a report even if the patient was discharged. Moreover, temporary active surveillance for social concern can influence the reported cases of carriers. The above-mentioned points can be considered as the reasons for the abnormal R/C ratio (exceeding 1) of EHEC and legionella^26^. Recently, Lyme disease and SFTS have been monitored under NIDS^27^, and physicians’ awareness has increased. An increase in R/C ratios should be interpreted carefully; if the R/C ratio has changed significantly, it is essential to evaluate factors that may result in changes in the reporting rate.

Pearson’s R represents the degree of correlation between the reporting- and claim-based surveillance. Even if the performance of the passive surveillance system was improved after the MERS-CoV outbreak, a low Pearson R-value for several diseases may suggest that either or both of the surveillance methods needs to be improved. Especially, considering that the diseases with low Pearson’s R were related to low incidence or episodic outbreaks, the conventional passive surveillance should remain as a cornerstone for monitoring low incidence infectious disease, because of time-consuming administrative process of the NHICS.

On the other hand, diseases with high Pearson R-value are a group in which claim-based surveillance may have the potential to complement report-based surveillance. In the post-MERS-CoV outbreak period, varicella showed a good concordance between the two systems, which may be due to its typical symptoms. The presence of typical symptoms may reduce the chance of misdiagnosis or unspecified diagnoses. With the same reason, a relatively high Pearson’s R was observed for the infectious diseases that have robust and easily accessible confirmatory examination for differential diagnosis, such as HAV, HFRS, leptospirosis, and malaria. In the case of scarlet fever and mumps, that have to be differentially diagnosed from certain infectious diseases, the incidence rates may not be accurate as per both NIDS- and NHICS, as it is not usual to perform a confirmatory examination for those diseases in Korea. However, this study demonstrated a good concordance, due to decreased hesitation in diagnosing and reporting relatively unique symptoms, even without a confirmed diagnosis. An increase in the population covered by the private health insurance is suspected to be a contributing factor. For infectious diseases complying with the above-mentioned conditions, the concordance between the two surveillance systems is high. Rather, NHICS can complement the low reporting rate of NIDS and its results can be more stable because it is not affected by the change in NIDS-associated reporting rate (due to MERS, temporary active surveillance, and newly implemented notifiable disease). Furthermore, considering the recent concern over the accuracy of NIDS due to an increased burden in data collection (e.g., the different diseases that need to be reported and the number of patients who need to be reported have increased), the applicability of NHICS data should be considered by public health officials.

As a linear regression analysis was performed to observe changes in R/C ratio over time, we observed an acceptable level of linearity from the results. This means that there is stability in the relationship between the two systems for the diseases. However, some infectious diseases presented no or poor linearity, which could be due to baseline noise in claims-based surveillance or those with recent low incidence. Considering the local epidemiology of mosquito-borne or tick-borne diseases in the ROK, it is evident that SFTS or Japanese encephalitis reported in January–March are not actual incidences but can be recognised as noise. In the observational period, the noise of claimed cases seems to be evident in the non-prevalent period of the respective diseases as the denominator of the R/C ratio increases. This baseline noise is believed to be prevalent throughout the claim-based surveillance period for whole subject infectious diseases in the present study. If information processing technology is developed to eliminate this noise, the usefulness of claim-based surveillance can be increased.

Another major problem regarding Korea’s infectious disease surveillance system is the lack of uniformity. This is because infectious disease surveillance systems are temporarily added whenever emerging or re-emerging infectious diseases are prevalent, rather than periodically, reforming the systems. Due to the healthcare system in Korea, there is a lack of laboratory-based surveillance systems. Most medical institutions in Korea are privately owned, and public hospitals account for only approximately 10%. Therefore, the laboratory is often owned by a private large hospital, and information from the laboratory is not shared. With certain emerging infectious diseases, national laboratory surveillance is carried out; however, information from private laboratories is not integrated even for infectious diseases with a high incidence.

Although several attempts have been made to introduce alternative surveillance systems at the global level^28^, they have not yet been widely used due to practical limitations, such as low accuracy and narrow coverage. In contrast, data from the NHIS database has become widely used health data by health researchers due to a rapidly advancing computerised system. Additionally, its cost burden is comparatively lower and it can be applied easily. The burden of data collection should be reduced to improve the accuracy of national surveillance systems. The case of scarlet fever and varicella^23,29^ in this study are good examples. The number of patients reported by the surveillance increased owing to the leniency in the reporting standard, which led to an increase in the burden of data collection, which, in turn, led to poor surveillance quality and inaccurate monitoring. The selection and concentration can be necessary with the assumption that the government has limited resources. For this, considering the aforementioned factors, NHICS can be considered as an alternative tool for reporting some infectious diseases.

However, there are notable disadvantages of the NHICS. Firstly, NHICS may generate wrong diagnosis codes due to up-coding. A study explained that medical providers may intentionally change diagnosis codes to prescribe certain type of drugs, such as antibiotics or other drugs that are covered by national health insurance for preventing further development of diseases. Secondly, claims and reviews are time-consuming processes that can be difficult to timely monitor for infectious diseases, as claims for the national health insurance are done monthly. Thirdly, NHICS does not obtain information to distinguish imported and domestic cases since the information is not necessary information required for health insurance.

Therefore, if we can compensate for the aforementioned drawbacks, we can establish a more effective surveillance system. For example, if a test result or drug use record is reported together, the accuracy of the monitoring system can be improved by preventing up-coding^12^. A system using medical records with available syndromic surveillance can also be established to supplement low timeliness, which is a disadvantage of claim-based surveillance^30^. The surveillance system should also be designed to identify the source of infections, whether it was acquired from abroad or domestic, through separate diagnosis codes. This claim-based surveillance may not be limited to infectious diseases. For example, an increase in certain diseases due to extensive exposure to novel chemicals can be better found in a claim-based surveillance. In addition, a traditional surveillance system could be supplemented to demonstrate the effectiveness of interventions, such as the national immunization program in the time series. We expect that countries that implement a new infectious disease surveillance system or NHIS can build a better surveillance system with the efforts mentioned above.

There have been attempts by many countries to use medical utilization data, such as claims data, as a means of infectious diseases surveillance. In the United States, claims data were used to respond to the persistent underreporting of Lyme disease and confirmed that claims data showed patterns similar to the national report-based surveillance and could solve the issue of under-reporting^31^. In Taiwan, claim-based surveillance was applied to varicella, and was less affected by the reporting rate^21^. In the case of major public health issues such as influenza, there have been attempts to use claims data more actively. In the United States, claims data have been used to monitor local and regional influenza activities^32^ and suggested that spatiotemporal relationships could be identified^33^. Claims data can also verify vaccination coverage. In Germany, claims data were used to identify the vaccination rate of major vaccines, which can be used as a basis for infectious diseases surveillance^34^.

However, claims data also have limitations. A comparison of claims-based surveillance and medical record-based surveillance for sepsis performed in the United States showed that for healthcare-associated infection or due to worsening of other disease conditions such as sepsis, claims based surveillance tended to be underreported rather than clinical data collected in electronic medical records^35^. These international studies suggest that claim based surveillance can be useful in infectious diseases with relatively low severity and high morbidity.

Our research has several limitations. First, incorrect diagnoses and reports can be found in both the NHIS and NHICS, which can lead to confusion in the interpretation. However, this limitation emphasises the need for NHICS as a complementary tool, paradoxically. In addition, studies to complement the inaccuracies of the two surveillance systems, such as using the “capture-recapture method”, are needed. Second, this study was conducted in single country. Third, each disease has different immune status or epidemics in each country. Therefore, the appropriate interpretation and application are necessary. Claim-based surveillance can be a good candidate for the surveillance of diseases that have a high incidence and are easily diagnosed by physicians (due to typical symptoms or highly accessible laboratory tests). Another problem to be solved for the application of claim-based surveillance is ‘information for action’, which requires timeliness. However, claims-based surveillance systems in Korea lack timeliness because of the billing behaviour of medical institutions, as most hospitals charge within one week of their patients discharge or visit, while some hospitals charge 1–2 months later. Therefore, no matter how fast the data processing is done, an inherent time delay will occur.

In conclusion, both report- and claim-based surveillance have unique advantages and disadvantages. The type of infectious surveillance methodology should be selected in consideration of the nature of infectious diseases. Certain methodology of surveillance systems may not be completely superior to another surveillance methodology. Although we expect claim-based surveillance to play an increasingly important role in monitoring infectious diseases, more active use of this system and studies on alternative surveillance systems are required for improving the performance of infectious diseases surveillance systems.

## Supplementary information

Supplementary table & figure

## Data Availability

The datasets generated during and/or analysed during the current study are available from the corresponding author on reasonable request.
