# Supplementary material for: Complementing conventional infectious disease surveillance with national health insurance claims data in the Republic of Korea
Source: Sci Rep. 2019 Jun 19;9:8750. doi: 10.1038/s41598-019-45409-3 (PMC6584579; doi:10.1038/s41598-019-45409-3)
Supplement: Supplementary file 1 — Supplementary table & figure [file 41598_2019_45409_MOESM1_ESM.pdf]

Complementing conventional infectious disease surveillance with national health insurance claims data in the Republic of Korea

Running title: Claim-based disease surveillance

Jaehun Jung[1,6^], Jae Hyoung Im[2^], Young-Jin Ko[3], Kyungmin Huh[4], Changgyo Yoon[5], Chulwoo Rhee[3], Young-Eun Kim[6], Dun-Sol Go[6], Arim Kim[6], Yunsun Jung[6], Munkhzul Radnaabaatar[6], Seok-Jun Yoon[6*]

[1] Department of Preventive Medicine, Gachon University College of Medicine, Incheon, Korea

[2]Department of Infectious Diseases, Inha University School of Medicine, Incheon, Republic of Korea

[3]Department of Preventive Medicine, Seoul National University College of Medicine, Seoul, Republic of Korea

[4]Department of Infectious Diseases, Samsung Medical Center, Sungkyunkwan University School of Medicine, Seoul, Republic of Korea

[5]Preventive Medicine Program, Graduate School of Public Health, Seoul National University, Seoul, Korea

[6]Department of Preventive Medicine, Korea University College of Medicine, Seoul, Republic of Korea

Corresponding author: Seok-Jun Yoon, Department of Preventive Medicine, Korea University College of Medicine, Seoul, Korea, E-mail: yoonsj02@korea.ac.kr [^]These authors contributed equally to this article

Supplementary Table 1. ICD-10 codes of the claim-based surveillance system and case classification of the National Infectious Disease Surveillance (NIDS)

Diseases	ICD-10[a]	Reporting criteria by case classification of the National Infectious Disease Surveillance (NIDS) (✓: Included, ×: Excluded)	
		Laboratory confirmed cases	Clinically suspected cases[c]
Brucellosis	A23, A23.0, A23.1, A23.2, A23.3, A23.8, A23.9	✓	✓
Cholera	A00, A00.0, A00.1, A00.9	✓	✓
Creutzfeldt-Jakob disease	A81.0	✓	✓
Dengue fever	A97, A97.0, A97.1, A97.2, A97.9, A90[a], A91[a]	✓	✓
Enterohemorrhagic Escherichia coli	A04.3	✓	✓
Viral hepatitis A	B15, B15.0, B15.9	✓	✓
Haemorrhagic fever with renal syndrome	A98.5	✓	✓
Japanese encephalitis	A83.0	✓	✓
Legionellosis	A48.1, A48.2	✓	✓
Leptospirosis	A27, A27.0, A27.8, A27.9	✓	✓
Lyme disease	A69.2	✓	✓
Malaria	B50, B50.0, B50.8, B50.9, B51, B51.0, B51.8, B51.9, B52 B52.0, B52.8, B52.9, B53, B53.0, B53.1, B53.8, B54	✓	×
Measles	B05, B05.0, B05.1, B05.2, B05.3, B05.4, B05.8, B05.9	✓	✓

Meningococcal meningitis	A39.0	✓	✓
Mumps	B26, B26.0, B26.1, B26.2, B26.3, B26.8, B26.9	✓	✓
Murine typhus	A75.2	✓	✓
Paratyphoid fever	A01.1, A01.2, A01.3, A01.4	✓	✓
Pertussis	A37, A37.0, A37.1, A37.8, A37.9	✓	✓
Q fever	A78	✓	✓
Rubella	B06, B06.0, B06.8, B06.9	✓	✓
Scarlet fever[b]	A38	✓	✓
Severe fever with thrombocytopenia	A93.8, A84, A84.8, A84.9	✓	✓
Shigellosis	A03, A03.0, A03.1, A03.2, A03.3, A03.8, A03.9	✓	✓
Scrub typhus	A75.3	✓	✓
Typhoid fever	A01, A01.0	✓	✓
Varicella	B01, B01.0, B01.1, B01.2, B01.8, B01.9	✓	✓

[a]HIRA uses the Korean Standard Classification of Diseases-6 (KCD-6), a variant of the ICD-10, which is only used in KCD-6.

[b]The criteria for reporting has been changed to include suspected cases since September 2012.

[c]Cases are designated temporarily as clinically suspected cases for early reporting and response. Once an appropriate laboratory test is performed to confirm the condition, this is then changed to a confirmed case

Supplementary Figure 1. Trend of 17 infectious diseases' reported, claimed cases, and reported cases per claimed cases ratio (R/C ratio) in the Republic of Korea, Jan 2010 - Jun 2017

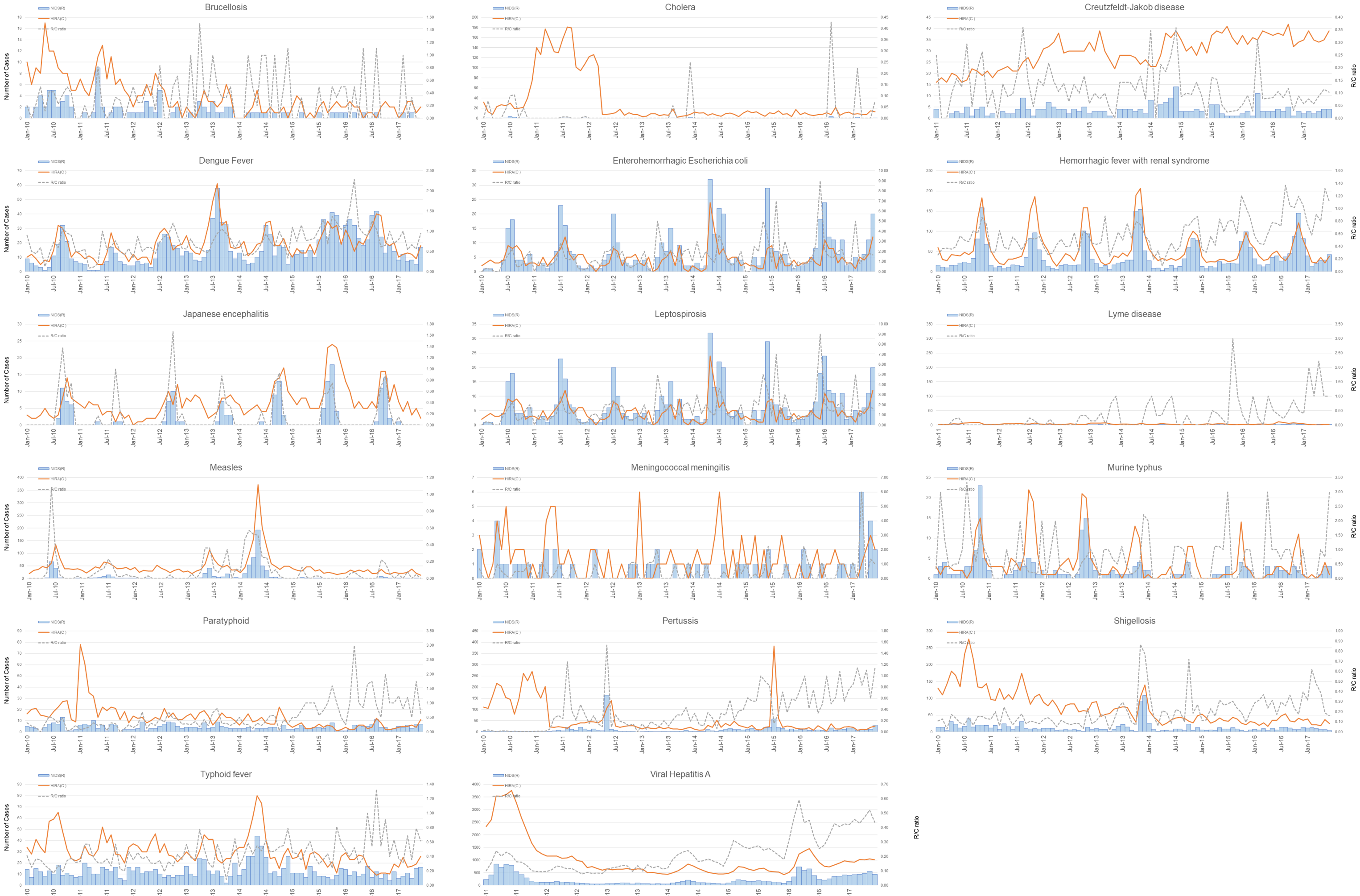
Brucellosis
Cholera
Creutzfeldt-Jakob disease
Dengue Fever
Enterohemorrhagic Escherichia coli
Hemorrhagic fever with renal syndrome
Japanese encephalitis
Leptospirosis
Lyme disease
Measles
Meningococcal meningitis
Murine typhus
Paratyphoid
Pertussis
Shigellosis
Typhoid fever
Viral Hepatitis A
Number of Cases
R/C ratio
NIDS(R)
HIRA(C)
R/C ratio
